# Candidate genes for field resistance to cassava brown streak disease revealed through the analysis of multiple data sources

**DOI:** 10.3389/fpls.2023.1270963

**Published:** 2023-11-03

**Authors:** Morag E. Ferguson, Rodney P. Eyles, Ana Luísa Garcia-Oliveira, Fortunus Kapinga, Esther A. Masumba, Teddy Amuge, Jessen V. Bredeson, Daniel S. Rokhsar, Jessica B. Lyons, Trushar Shah, Steve Rounsley, Geoffrey Mkamilo

**Affiliations:** ^1^ Cassava Breeding, International Institute of Tropical Agriculture (IITA), Nairobi, Kenya; ^2^ Cassava Breeding, Naliendele Agricultural Research Institute, Mtwara, Tanzania; ^3^ Cassava Breeding, Sugarcane Research Institute, Kibaha, Tanzania; ^4^ Cassava Breeding, National Crops Resources Research Institute (NaCRRI), Namulonge, Uganda; ^5^ Molecular and Cell Biology Department, University of California, Berkeley, Berkeley, CA, United States; ^6^ Bioinformatics, International Institute of Tropical Agriculture (IITA), Nairobi, Kenya; ^7^ Seeds & Traits R&D, Dow AgroSciences, Indianapolis, IN, United States

**Keywords:** lignin, phenylalanine ammonia-lyase, cinnamoyl-CoA reductase, eIF, PEPR1-related kinases, quantitative trait loci

## Abstract

Cassava (*Manihot esculenta* Crantz) is a food and industrial storage root crop with substantial potential to contribute to managing risk associated with climate change due to its inherent resilience and in providing a biodegradable option in manufacturing. In Africa, cassava production is challenged by two viral diseases, cassava brown streak disease (CBSD) and cassava mosaic disease. Here we detect quantitative trait loci (QTL) associated with CBSD in a biparental mapping population of a Tanzanian landrace, Nachinyaya and AR37-80, phenotyped in two locations over three years. The purpose was to use the information to ultimately facilitate either marker-assisted selection or adjust weightings in genomic selection to increase the efficiency of breeding. Results from this study were considered in relation to those from four other biparental populations, of similar genetic backgrounds, that were phenotyped and genotyped simultaneously. Further, we investigated the co-localization of QTL for CBSD resistance across populations and the genetic relationships of parents based on whole genome sequence information. Two QTL on chromosome 4 for resistance to CBSD foliar symptoms and one on each of chromosomes 11 and 18 for root necrosis were of interest. Of significance within the candidate genes underlying the QTL on chromosome 4 are Phenylalanine ammonia-lyase (PAL) and Cinnamoyl-CoA reductase (CCR) genes and three PEPR1-related kinases associated with the lignin pathway. In addition, a CCR gene was also underlying the root necrosis-resistant QTL on chromosome 11. Upregulation of key genes in the cassava lignification pathway from an earlier transcriptome study, including PAL and CCR, in a CBSD-resistant landrace compared to a susceptible landrace suggests a higher level of basal lignin deposition in the CBSD-resistant landrace. Earlier RNAscope^®^ *in situ* hybridisation imaging experiments demonstrate that cassava brown streak virus (CBSV) is restricted to phloem vessels in CBSV-resistant varieties, and phloem unloading for replication in mesophyll cells is prevented. The results provide evidence for the involvement of the lignin pathway. In addition, five eukaryotic initiation factor (eIF) genes associated with plant virus resistance were found within the priority QTL regions.

## Introduction

Cassava, a clonally propagated starchy root crop, is an important staple food and the third most important source of starch globally (Expert Market Research, 2023). Its relevance to the changing global environment with an increasing need for resilience against unpredictable and extreme weather patterns and the requirement to move away from plastics to more biodegradable solutions make cassava a crop for the future. Cassava and its products were estimated to provide on average 250 kcal/capita/day to the population in Africa in 2020 (FAOSTAT, 2023) making it an extremely important food source and trade commodity with tremendous potential for the future in Africa. Cassava is well adapted to the challenges of climate change, being naturally resilient to drought, high temperatures and low soil fertility. In Africa, two viral diseases, cassava mosaic disease (CMD), and cassava brown streak disease (CBSD), ravage cassava. These diseases are spread through the distribution of infected cuttings used as planting material and by whitefly (*Bemisia tabaci*) transmission (Maruthi et al., 2005). Resistance to these diseases is now among the highest breeding priorities, particularly in hotspot areas (Legg et al., 2014).

A relatively new viral disease, CBSD, caused by two species of Ipomovirus (Winter et al., 2010; Mbanzibwa et al., 2011), was, until 2003, restricted geographically to the East Africa coastal region where it was first reported by Storey (1936). After 2003, the disease began to spread in the Great Lakes region of East Africa (Alicai et al., 2007). The western frontier of the CBSD pandemic is now in eastern DR Congo, covering approximately 14% of the country (approx. 321,000 km^2^) with Haut-Katanga and Sud-Kivu being the latest provinces where CBSD has been detected (Casinga et al., 2021). CBSD is an important threat to West Africa (Legg et al., 2014). CBSD generally shows mild foliar symptoms but causes a brown corky necrosis in the storage roots, rendering them unusable. Nichols (1950) provides an excellent description of symptom expression.

Breeding for high levels of resistance is the priority approach for managing the impacts of CBSD. The first sustained effort in breeding for resistance to CMD and CBSD started in 1937 in Amani, northern Tanzania by the East African Agriculture and Forestry Research Organisation (EAAFRO) where crosses involving landraces and wild species were undertaken for addressing both CMD, as a priority, and CBSD (Jennings, 2003). Work on breeding for CBSD resistance continued from 1966, from the Naliendele Agriculture Research Institute, Tanzania after the Amani program closed in 1956 (Hillocks and Jennings, 2003). Here, sources of tolerance to CBSD focused on landraces from Tanzania and northern Mozambique, of which Nachinyaya, used in this study, is one. Since that time, new sources of resistance and even immunity to CBSV and Ugandan CBSV (UCBSV) have been uncovered in germplasm from South America (Sheat et al., 2019).

Phenotyping for CBSD is a challenging and expensive endeavour. Symptom expression can vary depending on growing conditions, as well as within and between roots (Hillocks and Jennings, 2003). Continued recycling of planting material over several years can lead to degeneration from the cumulative effects of disease (Shirima et al., 2019). All of these extends the time taken for accurate phenotyping. Locating quantitative trait loci (QTL) associated with CBSD resistance to facilitate either marker-assisted selection or adjust weightings in genomic selection has been a goal. Four bi-parental breeding populations, based on Tanzanian germplasm and conducted simultaneously, have been reported (Kapinga, 2017; Masumba et al., 2017; Nzuki et al., 2017; Garcia-Oliveira et al., 2020). Kayondo et al. (2018) and Somo et al. (2020) applied a Genome Wide Association Mapping (GWAS) approach to investigate the genetic architecture of CBSD and the potential of genomic selection through the assessment of genomic prediction in breeding for CBSD resistance.

Here we report on a fifth bi-parental mapping population, Nachinyaya × AR37-80, which was developed and phenotyped at the same time as four other populations; Namikonga × Albert (Masumba et al., 2017), NDL06/132 × AR37-80 (Kapinga, 2017), Kiroba × AR37-80 (Nzuki et al., 2017) and AR40-6 × Albert (Garcia-Oliveira et al., 2020). We compare the results with these populations and identify co-locating QTL for CBSD. This is viewed in relation to genetic relationships between the resistant parents gained from re-sequencing data (Bredeson et al., 2016), gene expression data from transcriptomic analysis of resistant and susceptible parents (Amuge et al., 2017), and imaging data from RNAscope^®^ *in situ* hybridisation (Sheat et al., 2020; Sheat et al., 2021) to identify candidate genes for CBSD resistance.

## Materials and methods

### Development of Nachinyaya × AR37-80 (NCAR) population

The CBSD-tolerant parent used to develop the NCAR population, Nachinyaya, is a landrace from northern Mozambique and south-eastern Tanzania. Its tolerance to CBSD root necrosis symptoms is well known, although it can show quite severe foliar symptoms. It is also susceptible to CMD (Hillocks et al., 1996; Hillocks et al., 2001; Thresh, 2003). The pollen parent, AR37-80, is a cross between a CMD-resistant line (C33) from IITA and CW259-42, the latter of which is a backcross of MTAI 8 (Rayong 60) and an interspecific cross between *M. flabellifolia* and CM 2766-5. It was developed through simple sequence repeat (SSR) marker-assisted selection, being positively selected for the CMD2 resistance locus and cassava green mite (CGM) resistance. It is, however, susceptible to CBSD (Blair et al., 2007; Okogbenin et al., 2012).

Pollinations were made by hand around midday according to Masumba et al. (2017) at the Sugarcane Research Institute (SRI) at Kibaha, Tanzania (6°46’52.22’’S, 38°58’25.05’’E). A flotation test was used to discard hollow seeds and select viable seeds which were then germinated in seed trays on benches in a screen house where temperatures were generally between 20°C to 30°C. Seedlings were transplanted into a field at Makutupora Agricultural Research Station (5°58’36.87’’S, 35°46’00.00’’E) in central Tanzania, an isolated, disease-free location. After one year the plants were ratooned and planted as the first phenotyping trial.

### Phenotyping

Phenotyping was conducted over three consecutive seasons, 2013/14, 2014/15 and 2015/16 in two CBSD hotspot locations in coastal Tanzania, Naliendele in the south (10°23’00.60’’S, 40°09’50.58’’E) and Chambezi (6°33’21.29’’S, 38°54’44.10’’E) on the central coast. Stakes were obtained from the disease-free multiplication site in Makutupora for the first year, then subsequently replanted from the harvested trial in each location for the second and third years, to facilitate the accumulation of viral load. Planting was done in January of each year to maximise disease pressure (Shirima et al., 2019). The site–season combinations were designated as experiments N1, N2 and N3 for Naliendele for 2013/14, 2014/15 and 2015/16 respectively, and experiments C1, C2 and C3 for Chambezi for the same seasons. Due to the large number of entries, an Alpha lattice design was used with incomplete blocks (Kashif et al., 2011). Five clonal plants were planted per plot at a spacing of 1 x 1 m in two replications. To increase the intensity and even distribution of disease pressure, CBSD-susceptible and infected plants with clear symptoms from surrounding farms were planted around each incomplete block. After data cleaning, a final population of 186 genotypes was used for analysis.

Scoring of foliar symptoms for CBSD was performed at 3 and 6 months after planting (MAP) in each experiment, on an individual plant basis, using the 1–5 scale (Hillocks and Thresh, 2000; Masumba et al., 2017). CBSD root necrosis was scored in N1, N2, C1, and C2 immediately after harvest (12 MAP), to avoid post-harvest physiological deterioration (Ravi et al., 1996). Root necrosis was not scored in C3 or N3. A maximum of seven randomly sampled roots per plant were assessed. Roots were cut using a knife or root-cutter (Kapinga, 2017) at equal intervals of 5 cm to expose the cross-section areas for CBSD severity assessment. A scale of 1–5 was used where 1 is no root necrosis, and 5 is over approximately 40% root necrosis (Masumba et al., 2017).

Shapiro-Wilk normality (SWILK) (Shapiro and Wilk, 1965), incorporated in the Genetic Analysis of Clonal F1 and Double Cross population (GACD v1.1) mapping software (Zhang et al., 2015), was used to determine the normality of the trait frequency distributions across the locations in both seasons.

### Genotyping

As cassava is an outcrossing species and parental stakes were partly derived from farmers’ fields, the integrity of the F1s was assessed for ‘off-types’ and ‘selfs’ (which are synonymous with outcross progeny between two clonal genotypes) using simple sequence repeat (SSR) markers. Leaf material from young plantlets was sampled for genomic DNA extraction according to a miniprep protocol modified from Dellaporta et al. (1983) based on Kapinga (2017). Initially 55 SSR primer pairs were used to screen the parents to identify polymorphic markers. Ultimately 14 of these, which produced unambiguous amplification products (NS911, SSRY100, -12, -151, -169, -171, -19, -38, -5, -51, -52 and -63) (Mba et al., 2001) were used to screen the entire population. Reaction conditions were according to Kawuki et al. (2013) and amplification products were resolved using capillary electrophoresis on an ABI 3730 and scored using GeneMapper v4.1 software. Results were used to identify true crosses.

DNA from true crosses were sent to UC Berkeley for genotyping-by-sequencing (GBS) (Elshire et al., 2011) with modifications (International Cassava Genetic Map Consortium, 2015; Masumba et al., 2017). Single-nucleotide polymorphisms (SNPs) were called against reference genome sequence version 5.1 (v5.1) and filtered for segregating loci as described by International Cassava Genetic Map Consortium (2015).

### Genetic linkage mapping and QTL analysis

A genetic linkage map was constructed from SNP data of true cross progeny in JoinMap^®^ v4.1. (van Ooijen, 2006). Initially, markers and individuals with more than 20% missing data were excluded, as were tetra-allelic loci and those with identical (ie. genetically redundant) segregation patterns. SNP marker data were coded according to the CP option in JoinMap v4.1 manual for outcrossing species. Bi-allelic SNPs provided segregation types ‘lmxll’, ‘nnxnp’ and ‘hkxhk’; and tri-allelic SNPs ‘efxfg’. The maximum-likelihood algorithm for cross-pollinated (CP) was used as is appropriate for outcrossing species in which both parents are heterozygous and the linkage phase is unknown.

A one-step genetic linkage map was generated with groups defined using a minimum Logarithm of Odds (LOD) of 5.0 with marker order being defined using the regression mapping algorithm (Wu et al., 2014) and Kosambi’s mapping function (van Ooijen, 2009). The linkage groups were named according to the corresponding chromosome as determined by the International Cassava Genetic Map Consortium (2015).

The mean trait score for each genotype across the replicates in each year and site was calculated and used for QTL mapping using inclusive composite interval mapping (ICIM) in Integrated Genetic Analysis Software of Clonal F1 and Double Cross Populations (GACD) version 1.1 (Yu et al., 2006; Zhang et al., 2015). Traits were CBSD foliar (3 and 6 MAP) and CBSD root necrosis. Based on the actual number of identified alleles in the two parents each marker locus was classified into four categories as described for GACD (Garcia-Oliveira et al., 2020). A significance threshold of LOD 3.0 was assigned manually. A LOD score of 3.0 or more is generally accepted as evidence of linkage as this implies that the likelihood in favour of linkage is 1000 times greater than the alternative. A QTL was considered ‘real’ when flanking markers of a significant QTL were consistent in two or more sites/seasons in any one population. Flanking markers, LOD scores, and adjusted percentage phenotypic variance explained (PVE%) are reported from the .QIC file.

QTL identifiers are prefixed with ‘q’ for ‘QTL’ followed by the trait abbreviation, ‘c’ for ‘chromosome’ and the number of the chromosome. If more than one trait QTL was identified per chromosome, then a point followed by a sequential number was used. A suffix ‘NC’ was added to specify that the QTL was identified in Nachinyaya. All QTL and marker positions are given in 5.1 of the cassava genome sequence, unless indicated.

Comparisons were made with QTL detected in four previously published bi-parental mapping populations, which were genotyped and phenotyped at the same time as the NCAR population (Kapinga, 2017; Masumba et al., 2017; Nzuki et al., 2017; Garcia-Oliveira et al., 2020), and QTL consistent across populations were highlighted. Candidate genes influencing resistance were selected from annotations from the Panther Classification System in combination with an extensive literature review of genes underlying the genomic positions of selected consistent QTL. This information was considered together with the gene expression data of these and related candidate genes from a time course transcriptomic experiment of UCBSV infected and uninfected Namikonga and Albert (Amuge et al., 2017).

## Results

Controlled pollination resulted in 1,216 seeds of which 49% (600 seeds) germinated after three months of storage to break dormancy. Of these, only 271 established well in the field at Makutupora. Initial quality control, to ensure the integrity of the population, did not reveal any selfs, but 11 off-types.

After excluding loci with significant deviation from Mendelian segregation as well as individuals and loci with a large amount of missing data, a data set of 199 individuals remained for genetic linkage mapping. A total of 2,887 SNPs were detected, of which 2,289 were mapped onto 18 linkage groups with a cumulative map length of 1802 centiMorgans (cM). The highest marker density was on chromosome 15 (average 0.47 cM between markers) and the lowest was on chromosome 13 (average 1.38 cM between markers) (**Supplementary File S1**). The average marker density was 1.27 SNPs per cM (**Supplementary File S1**). The genetic linkage maps of these populations formed the basis of the first high-resolution linkage map for cassava, which in turn formed the basis of the first physical map organized into chromosomes (International Cassava Genetic Map Consortium, 2015).

After consolidation and cleaning of genotyping and phenotyping data across sites and the removal of individuals with a large amount of missing data, 186 F1 individuals remained for QTL analysis. Overall mean scores for CBSD foliar symptoms, across both 3 and 6 MAP scores and all three growing seasons were higher in Chambezi (2.16) than Naliendele (1.19) (**Table 1**). Indeed, overall mean root necrosis scores were also higher in Chambezi (2.27) than in Naliendele (1.68). The same situation is reflected within years, with mean severity scores for CBSD root necrosis in Naliendele of 1.72 (N1) and 1.64 (N2) and Chambezi 2.2 (C1) and 2.34 (C2). For CBSD foliar symptoms, an increasing trend over the years in Naliendele 1.06 (N1), 1.18 (N2) and 1.34 (N3), and higher mean severity score in Chambezi, but not consistently increasing across years 2.4 (C1), 1.93 (C2) and 2.16 (C3). CBSD foliar severity scores were consistently higher at 6 MAP than 3 MAP in both locations (1.21 (6 MAP) compared to 1.18 (3 MAP) in Naliendele, and 2.51 (6 MAP) compared to 1.81 (3 MAP) in Chambezi) (**Table 1**).

**Table 1:** Basic statistics for CBSD root necrosis (CBSDRN) and CBSD foliar symptoms (CBSDF) from three seasons of phenotyping trials in Naliendele (N) and Chambezi (C).

Trait^a^	Experiment^b^	Mean (1–5 scale)	Variance	StdError	Skewness	Kurtosis	SWILK test	P-value
CBSDRN	N1	1.7182	0.4361	0.6604	1.3666	2.1902	0.8661	0.000^***^
	N2	1.6360	0.1510	0.3885	1.1347	2.7825	0.9112	0.000^***^
	C1	2.1962	0.3831	0.6189	0.9186	3.5837	0.8362	0.000^***^
	C2	2.3413	0.5797	0.7614	0.6587	1.4959	0.9171	0.000^***^
CBSDF	N1-3	1.0155	0.0082	0.0903	8.2033	78.119	0.1989	0.000^***^
	N1-6	1.1113	0.1506	0.3880	6.6301	56.3559	0.3476	0.000^***^
	N2-3	1.1777	0.1168	0.3418	4.0402	24.9493	0.5810	0.000^***^
	N2-6	1.1793	0.0693	0.2632	1.9729	3.8787	0.7097	0.000^***^
	N3-3	1.3527	0.1998	0.4470	2.0145	4.3965	0.7135	0.000^***^
	N3-6	1.3324	0.2094	0.4576	2.6699	8.8964	0.6705	0.000^***^
	C1-3	1.6361	0.1891	0.4348	0.3646	-0.4403	0.9478	0.000^***^
	C1-6	3.1625	0.9306	0.9647	-0.0122	-0.4980	0.9600	0.001^**^
	C2-3	2.0179	0.4060	0.6372	0.5850	0.7373	0.9376	0.000^***^
	C2-6	1.8401	0.6009	0.7752	1.3342	2.4359	0.8667	0.000^***^
	C3-3	1.7872	0.2590	0.5089	0.6328	1.5607	0.9159	0.000^***^
	C3-6	2.5242	0.3505	0.5921	-1.1533	1.1999	0.8476	0.000^***^

a CBSDRN—CBSD root necrosis, CBSDF—CBSD foliar symptoms.

b N1 = Naliendele 2013/14, N2 = Naliendele 2014/15, C1 = Chambezi 2013/14, C2 = Chambezi 2014/15, -3 = 3MAP, and -6 = 6MAP,

StdError = standard error, ** P ≤ 0.01; *** P ≤ 0.001.

### QTL associated with CBSD resistance

In the NCAR population, three consistent QTL associated with CBSD root necrosis resistance were detected on chromosomes 7, 11 and 12, all detected from data from C1 and C2 under high disease pressure (**Table 2**). A fourth region was detected on chromosome 18 in C1 only, characterised by two QTL, one quite specific (chrXVIII:6,327,979 to 6,801,336), the other encompassing the first (chrXVIII:4,212,438 to 8,916,735) (**Table 2**). Flanking markers, and distances between flanking markers, in the current version of the cassava reference genome assembly (v8.1; NCBI GenBank accession GCA_001659605.2, https://phytozome-next.jgi.doe.gov/info/Mesculenta_v8_1) (Bredeson et al. in preparation) can be found in **Supplementary File S2**. Logarithm of Odds (LOD) scores ranged from 2.64 (on chromosome 18) to 8.38 (on chromosome 11) from C2. The percentage of variance explained (PVE) ranged from 6.3 (on chromosome 7) to 32.4 (on chromosome 11).

**Table 2:** Consistent QTL identified in Nachinyaya × AR37-80 population for resistance to CBSD-induced root necrosis.

QTL name	Chr	Position (cM)	Trials in which the QTL was identified	Flanking markers (v5.1) (bp)		Parental effects			LOD	Adjusted PVE (%)
				Left marker	Right marker	F	M	FM		
qCBSDRNc7NC	7	26	C1	chrVII:4,715,293	chrVII:4,813,810	-0.1920	0.1275	0.1056	3.47	8.3
qCBSDRNc7NC	7	26	C2	chrVII:4,715,293	chrVII:4,813,810	-0.1995	0.1346	0.1336	3.48	6.3
qCBSDRNc11NC	11	27	C1	chrXI:4,383,294	chrXI:4,527,454	0.2605	-0.3863	-0.3637	4.41	31.7
qCBSDRNc11NC	11	27	C2	chrXI:4,383,294	chrXI:4,527,454	0.3384	-0.5399	-0.3221	8.38	32.4
qCBSDRNc12NC	12	37	C1	chrXII:6,934,834	chrXII:10,102,374	-0.0880	-0.0960	0.1203	3.04	7.5
qCBSDRNc12NC	12	40	C2	chrXII:6,934,834	chrXII:10,102,374	-0.1516	-0.1189	0.1116	3.85	7.8
qCBSDRNc18NC*	18	7	C1	chrXVIII:4,212,438	chrXVIII:8,916,735	-0.1024	-0.1063	0.1019	2.64	8.6
qCBSDRNc18NC*	18	28	C1	chrXVIII:6,327,979	chrXVIII:6,801,336	-0.0728	-0.1584	0.1767	2.84	12.2

* Although the LOD score is not above 3.0, due to consistencies with QTL in other populations (see below), this is worthy of inclusion.

Seventeen QTL that occurred in one or more site/season or scoring time-point were identified for foliar symptoms in the NCAR population. These occurred on 11 different chromosomes, i.e., 1, 4, 5, 6, 7, 8, 10, 11, 14, 17, and 18 (**Table 3**), with six QTL on chromosome 1. Data for individual sites/seasons can be found in **Supplementary File S3**. It is interesting to note that LOD and PVE scores were very high for Naliendele season 1 (2013/14). This may be due to the low disease pressure and non-normality of the data.

**Table 3:** QTL associated with CBSD foliar symptoms, scored at 3 and 6 MAP in Chambezi and Naliendele in three seasons, C1–3 and N1–3.

QTL name	Chr	Trials in which the QTL was identified*	Position (cM)	Flanking markers (v5.1) (bp)		LOD	Adjusted PVE (%)
				Left marker	Right marker		
qCBSDFc1NCa	1	C2^6^, N3 ^6^	30	chrI:3,985,072	chrI:4,383,262	9.39	44.4
qCBSDFc1NCb	1	N2 ^3^, C1^6^, N2^6^	33	chrI:4,658,167	chrI:4,815,462	13.06	37.4
qCBSDFc1NCc	1	N1 ^6^, N2^3^, N3^6^	57	chrI:9,703,189	chrI:10,030,347	25.28	61.2
qCBSDFc1NCd	1	N2 ^3^, N2^6^, C2^6^, N3^3^, N3^6^	74	chrI:13,330,388	chrI:14,450,997	10.14	37.6
qCBSDFc1NCe	1	N1 ^6^, N3^3^	102	chrI:19,342,967	chrI:19,919,971	19.58	62.6
qCBSDFc1NCf	1	N2^6^, N2^3^, C2 ^6^	106	chrI:21,287,026	chrI:21,864,131	4.39	28.8
qCBSDFc4NCa	4	N1 ^3^, N3^3^	82	chrIV:2,421,127	chrIV:2,421,144	22.56	70.9
qCBSDFc4NCb	4	N2 ^3^, N3^3^	4	chrIV:19,678,070	chrIV:20,065,474	9.17	41.5
qCBSDFc5NC	5	C1 ^6^, C2^3^	106	chrV:19,675,325	chrV:20,779,139	3.59	7.6
qCBSDFc6NC	6	C2^3^, C2 ^6^	73	chrVI:8,507,878	chrVI:8,507,925	6.17	16.6
qCBSDFc7NC	7	N1 ^6^, C2^6^, N3^3,6^	7	chrVII:261,026	chrVII:1,796,230	29.19	61.3
qCBSDFc8NC	8	C3^3^, N1 ^6^, N3^3^, N2^3^, N3^3^, C3^6^	37	chrVIII:12,759,725**	chrVIII:17,587,092	24.37	60.7
qCBSDFc10NC	10	C1^3^, N3^6^, N3 ^6^	66	chrX:14,023,601	chrX:14,620,013	9.49	40.3
qCBSDFc11NC	11	N3^3^, N3^3^, N1 ^3^	75	chrXI:13,715,995	chrXI:14,393,447	24.76	67
qCBSDFc14NC	14	N1 ^3^, C2^6^	70	chrXIV:14,795,105	chrXIV:14,835,447	25.36	70.9
qCBSDFc17NC	17	N3 ^6^, N1^6^, C2^3^	89	chrXVII:69,840	chrXVII:364,832	8.21	41.6
qCBSDFc18NC***	18	C2 ^3^, C3^3^	9	chrXVIII:4,212,438	chrXVIII:8,916,735	3.38	13.1

* The superscript denotes the time of scoring: ^3^, 3 MAP; ^6^, 6 MAP.

**QTL across the six trials indicated each had a QTL within this range, although the flanking markers were not consistent.

*** This QTL was significant for resistance to CBSD root necrosis in C1.

The LOD and PVE are reported for the trial which is underlined in the ‘Trial’ field.

### Comparison of QTL for CBSD across populations

**Figure 1:**
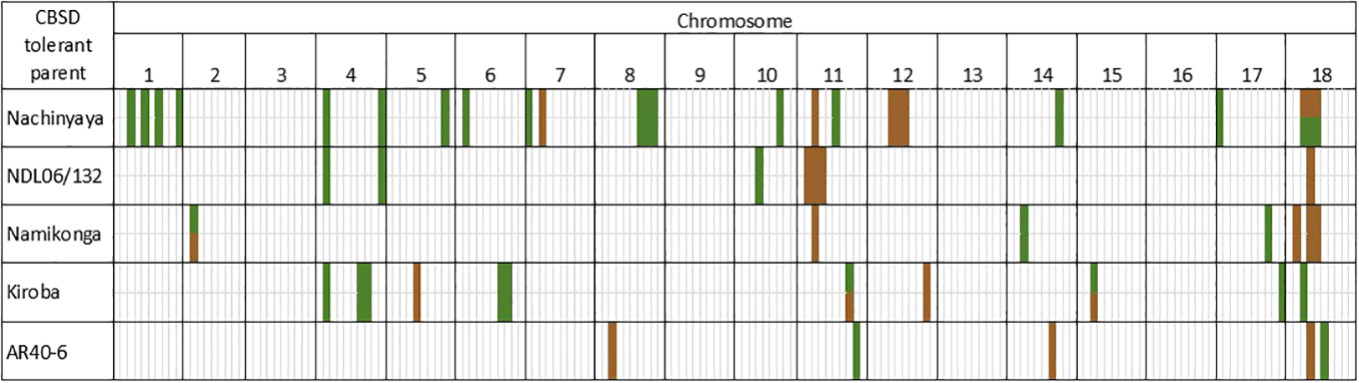
Schematic presentation of the positions of QTL in five bi-parental mapping populations developed for mapping resistance to CBSD foliar (green) and root necrosis (brown). Details of QTL can be found in **Supplementary File S4**.

QTL for both root necrosis and foliar symptoms, discovered in the NCAR population, were considered in relation to four other populations that were developed and phenotyped simultaneously i.e.: NDL06/132 AR37-80 (NDLAR) (Kapinga, 2017), Namikonga × Albert (NxA) (Masumba et al., 2017), Kiroba × AR37-80 (KAR) (Nzuki et al., 2017) and AR40-6 × Albert (ARAL) (Garcia-Oliveira et al., 2020). A total of 42 QTL were defined across populations for CBSD tolerance (**Supplementary File S4**); 10 for root necrosis, 29 for foliar symptoms and four for both, including one QTL on chromosome 8 from NCAR which covers a large region and encompasses four other QTL associated with either foliar symptoms or root necrosis and is thus not counted as a separate QTL. A schematic comparison of QTL for CBSD root necrosis and foliar symptoms can be found in **Figure 1** and compiled in **Supplementary File S4**. There are four chromosomal regions that are striking when viewing information across populations:

1. QTL on both arms of chromosome 4 appear important for resistance to CBSD foliar symptoms (**Table 4** and **Figure 1**). On the left arm, QTL from KAR (C1, C2) and NCAR (N1, N2) overlap from 2.4 to 3.4 Mbp and a QTL from NDLAR overlaps with NCAR (N1, N2) from 2.4 to 3.8 Mbp (C1, N1). This QTL has been designated qCBSDFc4_L.
2. On the right arm of chromosome 4, a QTL from NDLAR (N1) (19.4 to 19.5 Mbp) is very close to a QTL from NCAR (N2, N3) (19.7 to 20.1 Mbp) and has been designated as qCBSDFc4_R.
3. A series of QTL for CBSD-induced root necrosis from three populations (NDLAR, NCAR and NxA) between 3.6 and 7.3 Mbp on chromosome 11 are either overlapping, in series or close to one another and are here grouped as qCBSDRNc11 (**Figure 1** and **Table 5**). The first QTL from NCAR (N1 and N2) is from 3.6 to 4.3 Mbp with a second adjoining QTL from C2 from 4.3 to 5.3 Mbp. Encompassed within this second QTL is a QTL from NCAR (C1 and C2) and NxA (C1, C2). Adjoining this from 5.3 to 7.3 Mbp is another QTL from NDLAR in C1 and N1. The maximum amount of phenotypic variation explained is 32% in NCAR (C2).
4. The left arm of chromosome 18 is associated with resistance to CBSD foliar symptoms from around 4.2 to 8.9 Mbp in Nachinyaya, 5.8 to 6.0 Mbp in Kiroba and 10.0 to 10.9 Mbp in AR40-6 (**Table 6** and **Figure 1**). This region is designated qCBSDRNFc18.
5. For root necrosis resistance on chromosome 18, it appears there may be two QTL which are quite close to each other. Nachinyaya, Namikonga and AR40-6 all have QTL from 6.3 to 6.8 Mbp. NDL06/132 has a QTL for root necrosis overlapping this (from 6.7 to 9 Mbp) and Namikonga has a further QTL for root necrosis within this region from 8.6 to 8.9 Mbp (**Table 6** and **Figure 1**). These two overlapping QTL here are designated qCBSDRNc18a (6.3 - 6.8 Mbp) and qCBSDRNc18b (8.6 - 9.0 Mbp).

**Table 4:** Details of two regions with overlapping or closely positioned QTL on chromosome 4 for foliar symptoms across five populations phenotyped in the same sites and years.

QTL name^‡^	Resistant parent	Site/season (MAP*)	Flanking markers (v5.1) (bp)	LOD	PVE (%)
Composite QTL name qCBSDFc4_L					
qCBSDFc4KR	Kiroba	C1 & C2(3)	2,397,127–3,389,179	2.51–2.78	6.00–10.93
qCBSDFc4NCa	Nachinyaya	N2 & N1(3)	2,421,127–2,421,144	4.86–22.56	9.00–70.90
qCBSDFLc4a	NDL06/132	C1(9) & N1(6)	2,768,314–3,766,228	3.43–15.99	6.50–35.72
Composite QTL name qCBSDFc4_R					
qCBSDFLc4b	NDL06/132	C1(9)	16,481,626–17,222,649	2.77	5.10
qCBSDFLc4c		N1(3)	19,421,153–19,503,605	3.31	8.78
qCBSDFc4NCb	Nachinyaya	N3(6) & N2(3)	19,678,070–20,065,474	5.22–9.17	26.70–41.50

^‡^From original publication.

*Months after planting.

**Table 5:** Details of one region with overlapping or closely positioned QTL on chromosome 11 for CBSD-induced root necrosis (qCBSDRNc11) across five populations phenotyped in the same sites and years.

QTL name	Resistant parent	Site/season	Flanking markers (v5.1) (bp)	LOD	PVE (%)
Composite QTL name qCBSDRNc11_R					
qCBSDRNc11NDb*	NDL06/132	N1 & N2	3,559,047–4,301,373	3.19–5.6	11.52–11.93
qCBSDRNc11NDc*		C2	4,301,373–5,325,558	4.63	12.72
qCBSDRNc11NDa*		C1 & N1	5,325,558–7,316,171	2.86–12.65	14.75–17.8
qCBSDRNc11NC	Nachinyaya	C1 & C2	4,383,294–4,527,454	4.41–8.38	31.7–32.4
qCBSDRNc11Na	Namikonga	C1 & C2	4,502,175–4,527,454	3.35–3.81	5.2–7.6
qCBSDRNc11Nb		C2	4,527,454–4,617,294	7.5	17.8
qCBSDRNc11Nc		C1	4,617,294–4,760,631	3.6	5.3

*The names of the QTL have been modified from their original designation with an ND included after c11, to indicate the NDLAR population.

**Table 6:** Details of regions with overlapping or closely positioned QTL on Chromosome 18 for CBSD-induced root necrosis (qCBSDRNc18) and foliar symptoms (qCBSDFc18) across five populations phenotyped in the same sites and years.

QTL name	Resistant parent	Site/season	Flanking markers (v5.1) (bp)	LOD	PVE (%)
Composite QTL name qCBSDRNc18a and b					
qCBSDRNFc18NC	Nachinyaya	N2(6)	4,212,438–7,761,534	2.96	8.25
		C2(3), C3(3)	4,212,438–8,916,735	2.53–3.38	6.26–13.08
qCBSDRNFc18NC		C1(RN)	4,212,438–8,916,735	2.64	8.6
		C1(RN)	6,327,979–6,801,336	2.84	12.2
qCBSDFc18K	Kiroba	C2(6)	5,764,853–6,089,207	2.79	9.6
qCBSDRNc18Na	Namikonga	N2(RN)	6,320,754–6,502,253	3.31	8.21
		C1(RN)	6,327,979–6,801,336	2.84	12.2
qCBSDRNc18Nb		C2(RN)	8,650,285–8,943,971	5.1	6.49
qCBSDRNc18AR	AR40-6	N1(RN), C2(RN)	6,433,344–6,501,916	6.68–11.49	2.13–3.05
qCBSDFc18AR		C1(6), C2(6)	10,068,641–10,924,641	3.21–3.35	12–13
qCBSDRNc18ND	NDL06/132	C2(RN), N2(RN)	6,795,075–9,002,167	4.63–11.52	8.3–18.3

### Candidate genes associated with CBSD resistance

The QTL prioritised for selecting potential candidate genes were qCBSDFc4_L (2.4 - 3.8 Mbp), qCBSDFc4_R (19.4 - 20.1 Mbp), qCBSDRNc11 (3.6 - 7.3 Mbp) and qCBSDRNc18a (6.3 - 6.8 Mbp) and qCBSDRNc18b (8.6 - 9.0 Mbp) and contained 1,168 genes (**Supplementary File S5**). It was decided not to look further into foliar symptoms of Chromosome 18, as the QTL defined here was less precise and consistent. Gene families underlying these QTL with known association with viral resistance were selected from annotations from the Panther Classification System in combination with an extensive literature review (**Table 7**). Interestingly cinnamoyl-CoA reductase (CCR) genes involved in lignin synthesis were found within QTL regions on both chromosomes 4 and 11. These were also found to be highly upregulated in both infected and uninfected Namikonga compared to Albert in the study by Amuge et al. (2017). In fact, several genes in the lignin biosynthesis pathway were found to be upregulated in resistant Namikonga, compared to Albert (**Supplementary File S6**).

**Table 7:** Genes considered to have the highest likelihood of contributing to CBSD resistance contained within QTL on chromosomes 4, 11 and 18.

Trait	Right flanking (v5.1)	Left flanking (v8.1)	Right flanking (v8.1)	Gene ID (v8.1)	Region	Annotation
Chromosome 4						
Foliar	3,389,179	2,756,162	3,913,060	Manes.04G026700	3,132,187–3,137,031 (−)	Mitogen-activated protein kinase 3 (MAPK3)
Foliar	2,252,300	1,933,035	2,252,300	Manes.04G018000	2,203,816–2,207,282 (−)	Phenylalanine ammonia-lyase (PAL)
Foliar	15,734,008	30,208,991	31,029,727	Manes.04G103200	30,780,920–30,784,436 (−)	cinnamoyl-CoA reductase (CCR)
Foliar	3,766,228	3,195,542	4,403,548	Manes.04G028600	3,381,000–3,386,365 (−)	ribonuclease P/MRP protein subunit RPP1
Foliar	15,734,008	30,208,991	31,029,727	Manes.04G099100 Manes.04G099066 Manes.04G099300	30,355,304–30,362,981 (+) 30,365,861–30,377,729 (+) 30,379,086–30,383,919 (+)	Leucine-rich repeat receptor-like kinase PEPR1-related
Foliar	19,678,070	33,800,870	34,209,641	Manes.04G141500	34,006,333–34,010,108 (−)	Translation initiation factor 4A (EIF4A)
Foliar	15,734,008	30,208,991	31,029,727	Manes.04G102600	30,718,979–30,718,979 (−)	WRKY 40 transcription factor
Chromosome 11						
Root necrosis	5,325,558	6,600,507	8,959,420	Manes.11G063000	8,755,246–8,758,965 (−)	HOPW1-1-interacting1
Root necrosis	5,325,558	8,959,420	11,729,302	Manes.11G066500	9,469,194–9,471,365 (+)	WRKY 40 transcription factor
Root necrosis	4,527,454	6,600,507	8,959,420	Manes.11G064002	901,788–8,905,954 (+)	double strand RNA binding domain from dead end protein 1 DCL-4
Root necrosis	4,301,373	4,987,387	6,600,507	Manes.11G048500	5,229,033–5,235,934 (+)	Translation initiation factor 4A (EIF4AIII)
Root necrosis	4,617,294	6,984,296	7,146,789	Manes.11G058800	7,135,381–7,148,175 (+)	Translation initiation factor 3 (EIF3)
Root necrosis	4,527,454	6,861,781	6,886,880	Manes.11G056100	6,861,620–6,863,275 (+)	F18B13.21 protein-related
Root necrosis	4,617,294	6,886,880	6,984,296	Manes.11G056600	6,895,728–6,898,010 (−)	PPR repeat
Root necrosis	7,316,171	8,959,420	11,729,302	Manes.11G070900	10,223,822–10,236,933 (+)	Mitogen-activated kinase/threonine-protein kinase OSR1
Root necrosis	4,760,631	6,984,296	7,146,789	Manes.11G058000	7,005,871–7,014,518 (+)	Molecular chaperone HSP40/DNAJ-like protein
Root necrosis	7,316,171	8,959,420	11,729,302	Manes.11G065500	9,127,715–9,131,631 (+)	Cinnamoyl-CoA reductase (CCR)
Root necrosis	7,316,171	8,959,420	11,729,302	Manes.11G073900	10,660,113–10,667,824 (−)	DEAD BOX HELICASE-RELATED
Chromosome 18						
Root necrosis	6,801,336	8,032,346	8,535,185	Manes.18G091400	8,399,198–8,401,243 (−)	U6 snRNA-associated Sm-like protein LSm3 (LSM3)
Root necrosis	9,002,167	11,169,084	11,185,913	Manes.18G111600	11,169,084–11,185,913 (−)	Translation initiation factor 2D (EIF2D)
Root necrosis	8,916,735	5,339,282	11,217,402	Manes.18G072500	6,486,039–6,487,930 (−)	Translation initiation factor 5 (EIF5)
Root necrosis	8,916,735	5,339,282	11,217,402	Manes.18G071100	6,394,596–6,407,441 (−)	E3 ubiquitin Rab5/6 GTPase effector

## Discussion

A bi-parental mapping population was constructed from Nachinyaya (a landrace from northern Mozambique and southern Tanzania, known to have tolerance to CBSD root necrosis that has been durable for several decades, and susceptible to CMD) and AR37-80 (selected for the CMD2 gene and CGM resistance, but susceptible to CBSD (Blair et al., 2007; Okogbenin et al., 2012)). The final population size used for QTL analysis was 186 individuals. This population was phenotyped for CBSD root necrosis and CBSD foliar symptoms over three seasons (2013/14, 2014/15, and 2015/16) in two hotspot locations, Naliendele and Chambezi, Tanzania.

### QTL and candidate genes associated with CBSD resistance

A total of 42 QTL have been defined for tolerance to CBSD (**Supplementary File S4**) across five populations developed, phenotyped and genotyped at the same time as this population (NDL06/132 × AR37-80 (NDLAR) (Kapinga, 2017), Namikonga × Albert (NxA) (Masumba et al., 2017), Kiroba × AR37-80 (KAR) (Nzuki et al., 2017) and AR40-6 × Albert (ARAL) (Garcia-Oliveira et al., 2020). To prioritise QTL results for further investigation, they are considered in conjunction with other information (1) co-localisation across populations; (2) identity by descent (IBD) analysis of parents based on whole genome sequence information (Bredeson et al., 2016) and (3) introgression segments from *M. glaziovii* (Bredeson et al., 2016). Viewing results across populations, particularly with a knowledge of the relatedness of parents, and identifying QTL that occur in more than one population provides confidence in the validity of QTL.

Identity-by-descent analysis reveals the close relationship between the CBSD tolerance donor parents (Nachinyaya, Namikonga and NDL06/132) of three of the populations (NCAR, NxA and NDLAR), whereas the CBSD tolerant donors (Kiroba and AR40-6) of the other two populations are more independent (Bredeson et al., 2016; Nzuki et al., 2017). This has implications for interpreting the results. NDL06/132 and Nachinyaya appear to be full siblings, with Nachinyaya a full sibling of Albert, which in turn is a full sibling of TMEB117 (Bredeson et al., 2016). Namikonga is also closely related, with a parent–offspring relationship with TMEB117. TMEB117 is a Nigerian landrace that has been widely used in the IITA breeding program for poundability characteristics. TMEB117 was almost identical to TMEB693 (apart from CMD resistance) (Ferguson et al., 2019) and appeared to be widely distributed in Africa. This variety is possibly an early introduction to East Africa (Ferguson et al., 2019) and a parent in the Amani breeding program. This would explain the relationship between Nachinyaya, Albert and Namikonga. Namikonga is the Amani hybrid 46106/27, a third backcross of *M. glaziovii*, and mentioned by Hillocks and Jennings (2003) as one the best Amani hybrids. In Kenya, it is also known as Kaleso (Amuge et al., 2017). Interestingly NDL06/132 was thought to be a cross from female parent NDL90/34 and likely male parent Nachinyaya that was selected from a crossing block in Naliendele, southern Tanzania, although Bredeson et al. (2016) shows NDL06/132 and Nachinyaya to be full siblings. There was no *M. glaziovii* wild species introgression segment detected in either NDL06/132 or Nachinyaya (Bredeson et al., 2016). Many years ago, TMEB117 was reported to be ‘highly resistant’ (immune) to CBSV and, thus, maybe a source of CBSD resistance/tolerance (Stephan Winter, per. comm.).

In contrast, Kiroba is a landrace from around Dar es Salaam, which occasionally shows severe leaf symptoms of CBSV but no root symptoms, even under high disease pressure. The results from whole genome re-sequencing data, suggest Kiroba has a parent–offspring relationship with tree cassava (an *M. esculenta* – *M. glaziovii* hybrid) and is more related to West African and South American germplasm than East African germplasm (Nzuki et al., 2017). Introgression segments from *M. glaziovii* were identified by Nzuki et al. (2017) on chromosomes 1, 17 and 18. This, together with the fact that it has good yield and vigour, suggests it may be derived from the Amani breeding program. AR40-6 is a cross between a CMD-resistant line from West Africa and South American/Asian breeding lines. It clusters with the South American and West African germplasm (Nzuki et al., 2017) as opposed to East African germplasm.

In the NCAR population reported here, consistent QTL associated with resistance to CBSD root necrosis were detected on chromosomes 7, 11 and 12, all of which were identified within data from C1 and C2, cultivated under high disease pressure (**Table 2**). A fourth region was detected on chromosome 18 in C1 only, characterised by two QTL, one quite specific (chrXVIII:6,327,979–6,801,336), the other encompassing the first (chrXVIII:4,212,438–8,916,735) (**Table 2**). This also co-locates with QTL for foliar symptoms from this population. In addition, 17 significant QTL were associated with foliar symptoms. Here, we initially highlight QTL and chromosomal regions which are found in more than one population and provide possible explanations for this based on the relatedness of parents. We then highlight QTL that occur in only one population but either occur in many sites/seasons or, for other reasons, appear to warrant further investigation.

Two QTL on chromosome 4 are of interest; qCBSDFc4_L was associated with resistance to foliar symptoms in KAR (C1, C2) and NCAR (N1, N2) populations overlapping from 2.4 to 3.4 Mbp. This is noteworthy even though the donor parents of these populations are not particularly closely related. Of possibly greater interest is the QTL qCBSDFc4_R associated with foliar symptoms from NCAR and NDLAR (with CBSD tolerant donor parents Nachinyaya and NDL06/132, respectively). These parents are likely full sibs with an identity by descent (IBD) of 0.5411 based on whole genome sequence information (Bredeson et al., 2016). Neither Nachinyaya nor NDL06/132 have *M. glaziovii* introgression segments in their genome (Bredeson et al., 2016), implying that these QTL regions are derived from the *M. esculenta* genome.

Kayondo et al. (2018) using GWAS found many SNPs in high linkage disequilibrium, defining a QTL covering both arms of chromosome 4. This supports the evidence presented here. In contrast, Kayondo et al. (2018) suggest that this QTL is derived from an introgression segment from *M. glaziovii* and confirmed the presence and segregation of the introgressed genome segment in both panels using a set of diagnostic markers from *M. glaziovii*. Due to the high level of linkage disequilibrium at the QTL location, they do not highlight a single locus or loci as candidate gene(s) associated with CBSD foliar severity. Interestingly Somo et al. (2020), also using GWAS, detected a QTL on chromosome 4 (*QTL-cbsd4|cmd-1*), tagged by marker S4_24670203, which showed significant associations with both CBSD foliar symptoms and CMD resistance in the K4_cluster1 dataset. This is relatively close to the qCBSDFc4_R region (16,481,626–20,065,474 bp (v5.1).

Three populations with closely related CBSD donor parents (Namikonga, Nachinyaya and NDL06/132) all mapped the qCBSDRNc11 locus (**Figure 1** and **Table 5**). This locus is supported by evidence from 11 site/season combinations. The locus currently stretches over 3.7 Mbp from positions 3.6–7.3 Mbp, although the regions around 4.5 Mbp seem to be well supported with NCAR having a QTL covering only 144 kbp from 4,383,294 to 4,527,454 bp (6,683,896–6,886,880 bp in v8.1, a distance of 202,984 bp) from C1 and C2 and accounting for 32% of the variation in each site. In addition, NxA has a QTL covering just 25 kbp from 4,502,175 to 4,527,454 bp (6,861,782–6,886,880 bp in v8.1, 25,098 bp) from the same site/seasons but, in this case, accounts for an average of 6.4% of the phenotypic variation. There are no *M. glaziovii* introgression regions on chromosome 11 (Bredeson et al., 2016), so qCBSDRNc11 is likely to be derived from the *M. esculenta* genome. Given these data, we believe this region warrants further investigation.

In contrast to the study here, Kayondo et al. (2018) found 83 significant SNP markers on chromosome 11 associated with foliar symptoms at 3 MAP and 33 SNPs at 6 MAP, but around ~23 Mbp. They could not identify SNPs surpassing the Bonferroni threshold for CBSD root severity across two panels. However, analysis of the multi-location data for Panel 1 identified significant regions of CBSD association on chromosomes 5, 11 and 18 (−log10(P-value) > 6.5), which explained 8, 6 and 10% of the phenotypic variance, respectively. This supports the observations reported here regarding QTL on chromosome 11 and their association with CBSD root necrosis. In addition, Somo et al. (2020) identified a QTL associated with CBSD foliar symptoms, similar to that of Kayondo et al. (2018), but again these do not coincide with the QTL identified for root necrosis here.

A third region on chromosome 18 is more complex. A QTL occurs across a large region from 4.2 to 8.9 Mbp in NCAR and is associated with both foliar symptoms (C2, C3 and N2) and root necrosis (C1). The only other population showing a QTL associated with foliar symptoms within this region is KAR in C2 from 5.7–6.0 Mbp, albeit with a low LOD score of 2.79 with a PVE of 9.6%. It seems that there is more evidence for QTL associated with root necrosis in this region (qCBSDRNc18). Within the QTL region of NCAR (4.2–8.9 Mbp), there are two, more specific, regions defined by other populations. NxA, NCAR and the population ARAL with the unrelated donor parent AR40-6 have QTL from 6.3–6.8 Mbp, all associated with root necrosis in a total of five site/season environments. A neighbouring QTL from 6.8–9.0 Mbp in a further two populations (NDLAR (6.8–9.0 Mbp) and NxA (8.6–8.9 Mbp)) is associated with root necrosis in a total of three sites/seasons. Thus, all three related CBSD tolerant parents (NDL06/132, Nachinyaya and Namikonga) with good levels of tolerance to root necrosis have QTL for root necrosis between 6.3–9.0 Mbp on chromosome 18 (**Table 6** and **Figure 1**). Although this region is large, it appears to be genuine. ARAL shows a QTL for foliar symptoms from 10 to 10.9 Mbp in both C1 and C2, which may be of additional interest. There are no *M. glaziovii* introgression regions on chromosome 18 (Bredeson et al., 2016), so the QTL described here are likely to be derived from the *M. esculenta* genome. Kayondo et al. (2018) identified significant regions of CBSD root severity association from multi-location data for Panel 1 on 18 (−log10 (P-value) > 6.5), in addition to chromosomes 5 and 11, which explained 10% of the phenotypic variance.

Some QTL, not shared across populations, are worthy of mention and further investigation. The NCAR population, with donor parent Nachinyaya, apart from possessing the important qCBSDFc4_L, qCBSDFc4_R and qCBSDFc18 loci, contain other important QTL associated with foliar symptoms, including six QTL distributed across chromosome 1 and on chromosomes 5, 6, 7, 8, 10, 11, 14 and 17 (**Table 3**). Chromosome 1 has a large *M. glaziovii* introgression segment in some Amani-derived varieties (Bredeson et al., 2016), but interestingly *M. glaziovii* introgression segments were not found in Nachinyaya, and QTL associated with foliar symptoms were not found on chromosome 1 of other varieties with introgression segments. This indicates that foliar symptom resistance on chromosome 1 comes from the cultivated *M. esculenta*. In addition, QTL on chromosomes 7 (qCBSDRNc7NC) and 12 (qCBSDRNc12NC) were associated with root necrosis in C1 and C2 under high disease pressure, although these did have slightly lower PVEs compared to qCBSDRNc11NC and qCBSDRNc18NC. Somo et al. (2020) detected two QTL associated with both CMD and CBSD foliar symptoms on chromosome 12 (QTL-cbsd12|cmd-1 and QTL-cbsd12|cmd-2), the latter SNP tag S12_7929439 which is close to the QTL detected here for CBSD root necrosis 6,934,834–10,102,374 bp (v5.1), 8,250,631–12,995,530 bp (v8.1). It also overlaps with another QTL for CMD severity detected by Somo et al. (2020) at 6.3–8.7 Mbp (v5.1).

In Namikonga, a QTL on chromosome 2 named qCBSDRNFc2Nm from 3.5–3.6 Mbp (v5.1) was associated with foliar symptoms in all site/season combinations and root necrosis in both years in Naliendele and should be of particular interest. Somo et al. (2020) detected a QTL for root necrosis in the Tanzanian Ukiriguru GWAS population with SNP tagged at S2_9258334.

As Kiroba and AR40-6 are more distantly related to the other CBSD donor parents, they may possess unique QTL. For CBSD root necrosis and foliar symptoms, a small region (15.7–15.8 Mbp which corresponds with 26.956–27.058 Mbp in v8.1) on the other arm of chromosome 11 to the QTL from the Nachinyaya-NDL06/132-Naminkonga group, is implicated in the KAR population (qCBSDRNFc11KR). It is significant in C1, N1 and N2 and is worthy of further investigation (Nzuki et al., 2017). Somo et al. (2020) detected a QTL for CBSD foliar symptoms from between 22.88 and 22.94 Mbp in an Ukiriguru population.

In addition, a QTL on chromosome 15 (qCBSDRNFc15K) (4.2–4.7 Mbp) in the KAR population is significant for root necrosis in N2 and foliar symptoms in N1 and N2, but interestingly this was not detected under the higher disease pressure of Chambezi. Two further QTL related solely to root necrosis from the KAR population are also interesting; qCBSDRNc12K on chromosome 12 (16.4–17.3 Mbp), which is significant in C1, N1 and N2 and qCBSDRNc5K, although this is less consistent (Nzuki et al., 2017). The QTL found here does not coincide with that detected by Somo et al. (2020) for CMD and CBSD foliar symptoms. In ARAL, a QTL on chromosome 14 for root necrosis was detected when scoring was done using a 1–5 scale (qCBSDRNSc14AR) (12.1–13.9 Mbp) in N1, N2 and C2 and by area of necrosis (qCBSDRNAc14AR) (12.9–13.6 Mbp) in the same environments. This is worthy of further investigation and, for convenience, is designated here as qCBSDRNc14AR.

In summary, the polygenic nature of tolerance to CBSD root necrosis and foliar symptoms in the populations studied here is evident, as is their instability across environments. This was also noted by Kayondo et al. (2018). It appears that although tolerance to foliar symptoms and root necrosis are largely under different genetic controls, there are some regions of the genome which may influence both. It is recommended that the following two QTL are targeted, as first priority, for marker development for use in marker-assisted breeding for foliar symptom resistance; qCBSDFc4_L and qCBSDFc4_R; four for CBSD-induced root necrosis qCBSDRNc11, qCBSDRNc18 (which may also confer some tolerance to foliar symptoms), qCBSDRNc12K and qCBSDRNc14AR; and three QTL for both root necrosis and foliar symptom resistance: qCBSDRNFc2Nm, qCBSDRNFc11KR and qCBSDRNFc15K. Due to the large number of QTL detected across various mapping populations and panels, it is important that QTL should be mapped onto the most recent version of the cassava reference genome (Mbanjo et al., 2021).

### Candidate genes associated with CBSD resistance

A literature survey was carried out on the 1,168 genes contained within the priority QTL regions on chromosomes 4, 11 and 18 (**Supplementary File S5**) to determine those most likely to contribute to CBSD resistance (**Table 7**). Those considered the most significant are discussed below.

#### PAL and CCR

Of particular relevance are two genes, phenylalanine ammonia-lyase (PAL) (Manes.04G018000) and cinnamoyl-CoA reductase (CCR) (Manes.04G103200), found in QTL regions for foliar symptoms (chromosome 4). A second CCR (Manes.11G065500) is found within the QTL for root necrosis on chromosome 11. A second PAL gene (Manes.10G047500) was also identified on chromosome 10 (5,218,254–5,221,121 bp (v8.1)). CCR proteins operate downstream of PAL within the lignin biosynthetic pathway (Wadenbäck et al., 2008) and catalyse the production of several of its components (Yadav et al., 2020). We examined key genes of the cassava lignification pathway (Wang et al., 2023) using previously published transcriptomic data from Amuge et al. (2017). We found these to be strongly upregulated in the CBSD-resistant landrace Namikonga compared to the susceptible landrace Albert (**Supplementary File S6**) throughout the lignification pathway. This was the case in both uninfected and infected plants, strongly suggesting Namikonga has a higher level of basal lignin deposition. Wild cassava species, or those with introgression segments, tend to have more fibrous storage roots, commensurate with greater lignin content. Manes.11G065500 was upregulated 2.4x to 14.3x and 1.6x to 11.8x in Namikonga-infected and uninfected plants, respectively.

For long-distance and systemic infection, CBSVs, like other plant viruses, are translocated through the plant in both external and internal phloem vessels with the source-sink flow of photoassimilates (Graciano-Ribeiro et al., 2009; Nassar et al., 2010; Hipper et al., 2013; Sheat et al., 2021). Virus particles then pass from sieve elements of the phloem into the companion cells (SE–CC complex) to translocate to parenchyma and mesophyll cells, where they replicate. Resistance to long-distance movement is achieved either by preventing the virus from entering (loading) or exiting (unloading) the SE–CC complex (Rajamäki and Valkonen, 2009; Vuorinen et al., 2011). Sheat et al. (2021) demonstrated the extensive distribution of U/CBSV virus particles in phloem and non-phloem cells in leaves, stems and roots in CBSD susceptible varieties using RNAscope^®^ *in situ* hybridisation. In contrast, in a highly resistant variety from South America they demonstrated very few virus particles in the external phloem cells of the stem, with no accumulation, and an absence of viral particles in the parenchyma tissue. This indicates a restriction of movement, most likely in the unloading, of CBSV from the SE–CC to surrounding cells, thus preventing the virus from reaching sites of replication. In other clones, the virus was restricted to stems and roots or roots only, with organ-specific, variable phloem uploading implicated. This supports the observation here of different QTL for root and foliar CBSD symptoms implying that different mechanisms or conditions may exist for virus restriction in these tissues. Sheat et al. (2021) suggest that phloem restriction is a specific resistance response of the host since the viruses otherwise move and replicate extensively in susceptible cassava varieties.

CCR-mediated lignin synthesis and its contribution to pathogen resistance are reported in the case of fungal (Xu et al., 2011), bacterial (Liu et al., 2021) and viral resistance (tomato yellow leaf curl virus, Sade et al., 2015). It is thought that the increase in lignin deposition contributes to cell wall thickening, which acts as a physical barrier to unloading from the phloem to parenchyma cells at the onset of infection in resistant lines. However, the upregulation of components of the lignification pathway does not necessarily correlate with increased lignification (Kofalvi and Nassuth, 1995), suggesting further function of components of this pathway. CCR has also been shown to be an effector of Rac1, a small GTPase operating within plant defence signalling pathways and is an elicitor of the hypersensitive response to infection by Tobacco mosaic virus (Moeder et al., 2005; Kawasaki et al., 2006). The value of plant-derived lignin as an antiviral agent in non-plants has been an area of substantial research (reviewed in Ullah et al. (2022)). These studies have shown that lignin-derived material effectively inactivate a range of viral pathogens. The precise mechanism by which this is achieved is yet to be fully understood. However, mutants or overexpression lines within the lignin pathway suggest it performs an important role in pathogen resistance, both as a passive or active regulatory component of the immune response (Xu et al., 2011).

Although PAL operates upstream of CCR in the lignification pathway (thus polymorphisms within PAL are more likely to account for its up-regulation than CCR) the known functions and increased basal expression of CCR in Namikonga, seen in the Amuge et al. (2017) data, suggest it is a strong candidate for CBSD resistance

#### eIF

We identified eukaryotic initiation factor (elF) genes within QTL on chromosomes 4 (eIF4A), 11 (eIF4AIII, eIF3) and 18 (eIF5 and eIF2D). eIFs protein complexes or subunits are required to recruit mRNA to ribosomes for translation. This process can be co-opted by viruses for their own replication (Zhang et al., 2015). eIF4F complexes contain a 5´cap recognition subunit required for the correct placement of mRNA within the ribosome. Many viral transcripts, including those from members of *Potyviridae*, lack a 5´ cap but can utilise eIF complexes for translation through the binding of a cap-independent translation element (CITE) to an eIF4F subunit, eIF4G (Kneller et al., 2006). eIF4A functions as an RNA helicase, removing tertiary structures from mRNA prior to translation and is also likely to contribute to efficient viral RNA translation by substantially increasing the binding affinity of eIF4F to the CITE (Zhao et al., 2017). eIF4 and elF5 genes are known to be major contributors to plant virus recessive resistance, as minor sequence changes can prevent viral translation. This protection can be broad-ranging and mutations as minor as a single nucleotide have been shown to confer protection (Ling et al., 2009; Rodríguez-Hernández et al., 2012). While eIF4 has been a major focus of elF contribution to viral resistance (including the poly-A binding protein eIF4E in CBSD resistance (Shi et al., 2017)), more recently, eIF3 has been shown to be involved in barley yellow dwarf virus replication by facilitating binding viral 5′ UTR to the 40S subunit (Powell et al., 2022). We also note Manes.11G073900 within a QTL region on chromosome 11. This is annotated as a DEAD BOX HELICASE but has very high homology with other eIF4 proteins and may represent a third eIF on chromosome 11. A possible role of eIF2D in viral resistance has recently been proposed (Kim et al., 2023). It has been suggested that, upon viral infection, translation can be switched from the standard 5´cap dependent mechanism to one using non-AUG codons facilitated by eIF2A and/or eIF2D (Green et al., 2022). The cell uses this switch to prevent viral replication through interaction with eIF4 in the manner described above. However, positive-stranded RNA viruses are also able to utilise this mechanism and continue replication.

#### PEPR1

Chromosome 4 contains a cluster of three PEPR1-related kinase genes. PEPR1/2 proteins act as peptide receptors as part of an innate immunity activation network. *Arabidopsis* plants with disruptions in this pathway show reduced root callose and lignin deposition and are more susceptible to infection by *Pseudomonas syringae* (Jing et al., 2023). AtPEPR1 is one of several proteins which can act as co-receptors within this network with BRASSINOSTEROID INSENSITIVE 1-associated receptor kinase 1 (BAK1). Mutant forms of AtBAK1 show increased susceptibility to a range of viral infections, while AtPEPR1 mutants do not show variation in viral load (Yang et al., 2010; Kørner et al., 2013). AtPEPR1/2 gene expression is linked with increased salicylic acid (SA) production, a major factor in plant viral defence (reviewed in Alazem and Lin, 2015). Additionally, AtPEPR1 is induced upon wounding by feeding insects, including whitefly (Wang et al., 2019), presumably to prime the immune response against invasive microbes. PEPR1 studies have been largely restricted to *Arabidopsis*, and a role in viral defence in other species should not be ruled out.

#### WRKY

The data contained a single WRKY 40 transcription factor gene underlying the QTL on chromosome 4 and two WRKY 25 transcription factor genes on chromosome 18. The WKRY gene family is manifold, performing a variety of functions, including regulation of biotic stress response (Chen et al., 2017; Jiang et al., 2017). In *Arabido*psis, WKRY40 and WKRY25 proteins are negative regulators of key resistance genes and mutants show decreased susceptibility to *P. syringae* infection (Zheng et al., 2007; Pandey et al., 2010). Notably, transcriptional profiling indicates a possible role for another WKRY40 homologue in resistance to the South African cassava mosaic virus (Freeborough et al., 2021).

#### MAPK3

We identified a mitogen-activated protein kinase 3 (MAPK3) gene on chromosome 4 (Manes.04G026700). MAPK proteins are a component of signalling cascades with several known functions, including being strongly associated with SA and jasmonic acid (JA) mediated immunity. In tomato, SlMAPK3 enhances tolerance to tomato yellow leaf curl virus (TYLCV) and is upregulated in infected plants (Li et al., 2017). Although cassava contains multiple MAPK3 genes, Manes.04G026700 has the highest peptide similarity (84%) to SlMAPK3. In a variety of plant species, endogenous application of JA increases resistance to viral infection and increases transcription of MAPK genes (Shang et al., 2011).

#### HOPW1-1

*M. esculenta* HOPW1-1-interacting1 (MeWIN1) protein is a member of the plant-specific AP2/EREBP transcription factor family. MeWIN1 is a homologue of AtSHN1/WIN1, which contributes to infection resistance by various fungi by regulating the induction of pathogenesis-related and redox-related genes (Sela et al., 2013). Besides, WIN1 possibly regulates SA in Arabidopsis through interaction with hopW1-1 effector proteins in response to *Pseudomonas syringae* infection (Lee et al., 2008). In cotton, GhWIN2 positively regulates JA biosynthesis but negatively influences SA biosynthesis (Li et al., 2019). WIN1 is strongly linked with cuticle formation, and increased expression has been shown to enhance the physical barrier against leaf-feeding insects and, by extension, their viral load. Beyond this function, WIN1 has been shown to perform multiple roles including pathogen defence and hormone regulation.

#### DCL-4

Manes.11G064002, annotated as a DICER-LIKE 4 (DLC-4) protein-encoding gene, was strongly up-regulated in Namikonga, five days post-infection, compared to Albert (Amuge et al., 2017). DCL-4 proteins form part of the plant RNAi immunity system and are involved in virus-induced RNA silencing (VIGS) by mediating the biogenesis, at the site of viral infection, of small interfering RNAs. These can be triggered upon infection and act non-cell autonomously to enable viral defence mechanisms in uninfected tissue (Garcia-Ruiz et al., 2010).

#### LSM3

Members of the LSM family of proteins are involved in eukaryotic mRNA turnover through de-adenylation and de-capping mechanisms. Positive-stranded RNA viruses require host translation to produce RNA replication proteins which, in turn, replicate viral RNA by recruiting it from the host translation mechanism (Chen and Ahlquist, 2000; Schwartz et al., 2002). To distinguish viral from host RNA, Brome mosaic virus has been shown to utilise the mRNA processing function of LSM proteins, and LSM deficiency blocks viral RNA translation (Noueiry et al., 2003).

#### Rab5/6 GTPase effector protein

Rab GTPases are present on the surface of organelles where they act as recognition and binding platforms for intracellular proteins but are also widely involved in viral infection (reviewed in Spearman, 2018). Positive-stranded RNA virus replication utilises membrane-bound viral replication proteins as well as host membrane proteins, including Rab5 and Rab5 effectors (Stone et al., 2007).

#### LRR

The most frequent class of plant resistance genes (R genes) are denoted as NBS-LRR, which usually contain both a nucleotide-binding site domain and a leucine-rich repeat domain (McHale et al., 2006). The LRR domain is thought to be a major determinant of pathogen recognition specificity (Collier and Moffett, 2009). A total of 228 NBS-LRR type genes and 99 partial genes (without the NBS domain) were located on v4.1 of the casava genome sequence (Lozano et al., 2015). There were several LRR proteins underlying all target QTL investigated here, with 11 such genes within a 1.28 Mbp region underlying the QTL on chromosome 11. Although Kayondo et al. (2018) identified a cluster of NBS-LRR genes on chromosome 11 through genome-wide associated mapping and genomic selection, this region was outside of the QTL region investigated here. Neither Maruthi et al. (2014) nor Lozano et al. (2015) found any significant differential expression of NBS-LRR genes in cassava infected with CBSV; however, NBS-LRR genes were implicated in resistance to cassava anthracnose disease (Utsumi et al., 2016). Zhang et al. (2022) selected four NBS-LRR genes from the transcriptome databases of Lozano et al. (2015) and Utsumi et al. (2016) for further investigation and found that they were significantly induced by *Xanthomonas axonopodis* pv. *manihotis* (Xam) and salicylic acid treatment. Of these, the MeLRR1 gene (Manes. 11G053000.1) is within the QTL region on chromosome 11 investigated here.

## Conclusions

A total of 42 QTL have been defined across five bi-parental mapping populations, developed, genotyped and phenotyped predominantly to detect QTL associated with tolerance to CBSD in largely Tanzanian germplasm. This indicates that this source of tolerance to CBSD root necrosis and foliar symptoms is quantitative. Most QTL are specific for either root necrosis or foliar symptoms, indicating they are largely independently controlled and explaining field (Kayondo et al., 2018; Garcia-Oliveira et al., 2020) and imaging (Sheat et al., 2021) observations. Four QTL are highly supported by cross-population evidence; two on chromosome 4 associated with foliar symptoms, and two on chromosomes 11 and 18 associated with root necrosis. Tolerance to root necrosis does not appear to come from wild species but from TME117 or a similar genotype. Additional population-specific QTL were highlighted for further investigation in four populations. This provides opportunities for QTL stacking. Evidence from the co-location of QTL, parental relationships, gene expression from transcriptome analysis and imaging reports highlight several candidate genes for CBSD resistance. These include PAL and CCR genes involved in the lignin synthesis pathway, in resistance to both foliar CBSD symptoms (chromosome 4) and root necrosis (chromosome 11) and the PEPR1-related kinases shown to be involved with lignin synthesis and virus resistance. The involvement of the lignin pathway can explain, in part, the previously reported restriction of virus particles to phloem tissues in resistant varieties. Further research should endeavour to validate these results and determine whether they are applicable to other resistant varieties, particularly those of South American origin. The presence of eIF underlying QTL should be of interest in the case of Potyvirus resistance. These genes provide good candidates for validation and the development of molecular markers for marker-assisted breeding. Alternatively, due to the polygenic nature of these traits, a genomic selection approach to breeding may be more appropriate.

## Data availability statement

The original contributions presented in the study are included in the article/**Supplementary Material**. Further inquiries can be directed to the corresponding author.

## Author contributions

MEF: Conceptualization, Data curation, Formal Analysis, Funding acquisition, Investigation, Methodology, Project administration, Supervision, Writing – original draft, Writing – review & editing. RPE: Data curation, Formal Analysis, Investigation, Methodology, Writing – review & editing. AL-O: Data curation, Formal Analysis, Investigation, Writing – review & editing. FK: Data curation, Formal Analysis, Investigation, Writing – review & editing. EM: Investigation, Writing – review & editing. TA: Investigation, Writing – review & editing. JB: Investigation, Writing – review & editing. DR: Conceptualization, Funding acquisition, Project administration, Supervision, Writing – review & editing. JL: Investigation, Writing – review & editing. TS: Formal Analysis, Writing – review & editing. SR: Conceptualization, Funding acquisition, Methodology, Writing – review & editing. GM: Conceptualization, Funding acquisition, Project administration, Writing – review & editing.
