# Supplementary material for: Candidate genes for field resistance to cassava brown streak disease revealed through the analysis of multiple data sources
Source: Front Plant Sci. 2023 Nov 3;14:1270963. doi: 10.3389/fpls.2023.1270963 (PMC10655247; doi:10.3389/fpls.2023.1270963)
Supplement: Supplementary file 6 [file Image_1.pdf]

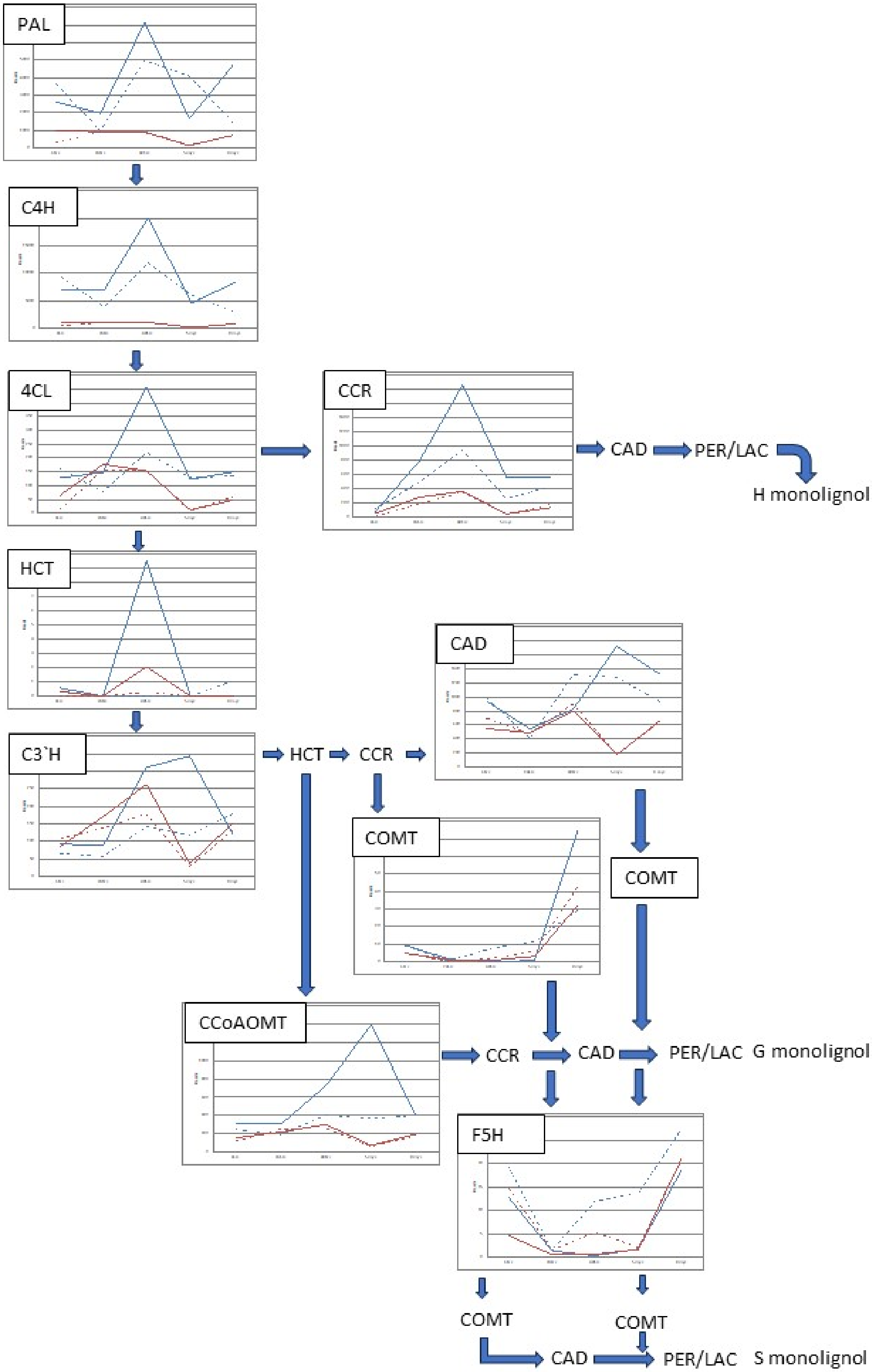
PAL
C4H
4CL
CCR
CAD
PER/LAC
H monolignol
HCT
CAD
C3`H
HCT
CCR
COMT
COMT
CCoAOMT
CCR
CAD
PER/LAC
G monolignol
F5H
COMT
COMT
CAD
PER/LAC
S monolignol

Supplementary file S6: Relative expression from normalised data of lignin biosynthesis enzymes from Albert and Namikonga landraces. Expression in Namikonga (blue) is higher than Albert (red) in both uninfected (unbroken lines) and infected (dashed lines) plants throughout the pathway. Data from Amuge *et al.* (2017) and Zhao and Dixon (2011). Y-axis: normalised read count, X-axis, time points.

Abbreviations: PAL (l-phenylalanine ammonia-lyase, cassava4.1_002591m.g, Manes.04G018000), C4H (cinnamate 4-hydroxylase, cassava4.1_005978m.g, Manes.18G126900), 4CL (4-hydroxycinnamate: CoA ligase, cassava4.1_005014m.g, Manes.04G095300), HCT (hydroxycinnamoyl CoA shikimate hydroxycinnamoyl transferase, cassava4.1_034337m.g, Manes.10G050800), C3'H (p-coumaroylshikimate 3'-hydroxylase, cassava4.1_005910m.g, Manes.08G063400), CCoAOMT (caffeoyl CoA 3-O-methyltransferase, cassava4.1_014783m.g, Manes.07G075700), CCR (cinnamoyl CoA reductase, cassava4.1_011283m.g, Manes.11G065500), COMT (caffeic acid 3-O-methyltransferase, cassava4.1_030204m.g, Manes.18G083570), F5H (ferulic acid 5-hydroxylase, cassava4.1_025966m.g, Manes.02G100800) and CAD (cinnamyl alcohol dehydrogenase, cassava4.1_010625m.g, Manes.16G057118).

Morag E. Ferguson, Rodney P. Eyles, Ana Luísa Garcia-Oliveira, Fortunus Kapinga, Esther A. Masumba, Teddy Amuge, Jessen V. Bredeson, Daniel S. Rokhsar, Jessica B. Lyons, Trushar Shah, Steve Rounsley, Geoffrey Mkamilo

Candidate genes for field resistance to cassava brown streak disease revealed through the analysis of multiple data sources.

Frontiers in Plant Science
